# Patients with Different Stages of Chronic Kidney Disease Undergoing Intravenous Contrast-Enhanced Computed Tomography—The Incidence of Contrast-Associated Acute Kidney Injury

**DOI:** 10.3390/diagnostics12040864

**Published:** 2022-03-30

**Authors:** Ming-Ju Wu, Shang-Feng Tsai

**Affiliations:** 1 Division of Nephrology, Department of Internal Medicine, Taichung Veterans General Hospital, Taichung 407, Taiwan; wmj530@gmail.com; 2 School of Medicine, Chung Shan Medical University, Taichung 402, Taiwan; 3 Rong Hsing Research Center for Translational Medicine, Institute of Biomedical Science, College of Life Science, National Chung Hsing University, Taichung 402, Taiwan; 4 Graduate Institute of Clinical Medical Science, School of Medicine, China Medical University, Taichung 404, Taiwan; 5 Department of Post-Baccalaureate Medicine, College of Medicine, National Chung Hsing University, Taichung 402, Taiwan; 6 Department of Life Science, Tunghai University, Taichung 407, Taiwan; 7 School of Medicine, National Yang-Ming University, Taipei 112, Taiwan

**Keywords:** acute kidney injury, dialysis, mortality, chronic kidney disease, intravenous contrast medium, computed tomography

## Abstract

Introduction: Iodinated contrast medium (CM) is the third most common cause of acute kidney injury (AKI). However, the association is poorly known between the definitions of AKI between different stages of chronic kidney disease after intravenous CM administration. Methods: The dataset, covering a period of ~15 years (1 June 2008 to 31 March 2015), consisted of 20,018 non-dialytic adult patients who had received intravenous injections of non-ionic iso-osmolar CM, iodixanol, for enhanced computed tomography imaging. Contrast-associated AKI (CA-AKI), dialysis-required AKI, and mortality were analyzed. Results: A total of 12,271 participants were enrolled. CA-AKI increased significantly starting from stage 3A onward (*p* < 0.001). In summary, incidences of CA-AKI against different levels of chronic kidney disease were as follows: stage 1 (8.3%) = stage 2 (6.7%) < stage 3A (9.9%) < stage 3B (14.3%) < stage 4 (20.5%) = stage 5 (20.4%). The incidences of dialysis within 30 days were as follows: stage 1 (1%) = stage 2 (1.4%) = stage 3A (2.7%) < stage 3B (5.7%) < stage 4 (18%) < stage 5 (54.1%). The prediction of dialysis was good based on the baseline serum creatinine > 1.5 mg/dL (72.78% of sensitivity, 86.07% of specificity, 0.851 of area under curve) or baseline estimated glomerular filtration rate ≤ 38.49 mL/min/1.732 m^2^ (70.19% of sensitivity, 89.08% of specificity, 0.853 of area under curve). In multivariate Cox regression analysis model for CA-AKI, independent risk factors were stage 4 chronic kidney disease (*p* = 0.001) and shock (*p* = 0.001). Conclusion: Baseline serum creatinine and estimated glomerular filtration rate were good predictors for dialysis-required AKI. CA-AKI increased significantly since stage 3A chronic kidney disease. Stage 4 and 5 chronic kidney disease have the same risk for CA-AKI, but stage 5 chronic kidney disease has markedly higher risk for dialysis.

## 1. Background

Iodinated contrast medium (CM) is the third most common cause of acute kidney injury (AKI) [1], which increases the risks of dialysis and mortality. The most common cause of contrast-induced nephropathy (CIN) is pre-existing chronic kidney disease (CKD). Since 2008, a number of studies have indicated that the severity of CKD is a risk factor for the development of AKI [2,3]. Specifically, CIN occurs following CM exposure in 8% of patients with estimated glomerular filtration rate (eGFR) lying between 45 to 60 mL/min/1.73 m^2^, 13% with eGFR between 30 to 45 mL/min/1.73 m^2^, or 27% with eGFR < 30 mL/min/1.73 m^2^ [4,5,6]. Only post-contrast renal outcome has been reported when eGFR falls below 30 mL/min/1.73 m^2^_._ Little is known about the AKI, dialysis and mortality after CM administration in patients with further severe CKD (i.e., stages 4 and 5) [7,8,9]. Such studies on CKD and CIN when also reported is based on a small number of patients [10].

CIN, classically defined by an increase in serum creatinine (SCr) levels by 25% or >44 μmol/L that occurs within 48–72 h after intravenous (IV) CM administration [11], was replaced recently by contrast-associated AKI (CA-AKI) based on the definition of AKI (Kidney Disease Improving Global Outcomes (KDIGO) practice guideline [12]), which broadened the period of renal dysfunction from 48–72 h to 7 days. Most earlier studies on post-contrast renal impairment were based on the classical definition of CIN [11] rather than the newer CA-AKI. Therefore, the study between CA-AKI (occurs within 7 days) after CM administration is largely limited.

The risk of CIN due to IV CM maybe overstated [13,14]. Clinicians used high-osmolality CM in early studies, but low-osmolality CM agents with much less CIN [15] were used instead in the current era. The Contrast Media Safety Committee (CMSC) of the European Society of Urogenital Radiology (ESUR) recommended an eGFR < 45 mL/min/1.73 m^2^ before an IV CM application and an eGFR < 60 mL/min/1.73 m^2^ before an intra-arterial (IA) CM administration is considered a risk factor for CIN [13,16,17]. More chances of CIN can likely occur with IA CM than with IV CM. The literature regards incidences of CIN that occur typically following cardiac angiography, with lower risks of CIN after IV injection. Most previous studies are on IA CM-related CIN for patients with either cardiac catheterization or percutaneous coronary intervention. IV CM administration is not associated with higher risks of CIN, emergent dialysis, and short-term mortality in another study [18]. Similarly, a retrospective cohort study in 2017 showed that IV CM from contrast-enhanced CT (CCT) (with iso- or low- osmolar agents) does not associate with more incidences of CIN [10]. On the contrary, IV CM was found to be associated with CIN in two other studies [19,20]. The association remains controversial between IV CM administration from CCT and CIN. We here aimed to study the association between IV CM from CCT and renal dysfunction in patients with different baseline renal functions, using a large database to determine the association between different stages of CKD and renal impairment (defined by CA-AKI instead of CIN) in patients undergoing IV CM (iso-osmolar CM, 100 mL) from CCT.

## 2. Materials and methods

### 2.1. Study Design and Patient Population

We conducted this retrospective historical cohort study on a database at our hospital (Taichung Veterans General Hospital) as recorded from 20,018 adult patients without dialysis receiving IV non-ionic and iso-osmolar CM (iodixanol (Visipaque, Chicago, IL, USA)) for enhanced CT during a period of ~15 years (1 June 2008 to 31 March 2015). All data were from electronic medical record system in our institute. The data for each patient included baseline SCr within two days before CCT. Data were employed to determine the post-CCT association between baseline stages of CKD and renal outcome (CA-AKI [12], dialysis within 30 days, and mortality). Excluded participants were patients with pre-existing AKI (defined according to KDIGO practice guideline [12]), with recent exposures to CM over the preceding 30 days, volume of CM not equal to 100 mL (usual volume of CM for CCT), not available baseline SCr within the two days before CCT, and not available post-contrast SCr within one week after CCT.

The institute review board of Taichung Veterans General Hospital approved this study (IRB TCVGH No: F15059). The entire procedures were performed in accordance with the relevant guidelines and regulations. Informed consent was waived due to the data evaluation nature of the study. We had no official protocol for the prevention of CIN in our institute, such as IV normal saline hydration, sodium bicarbonate infusion, and N-acetylcysteine during the period of this study.

### 2.2. Baseline Information Retrieval and Definition

Baseline stages of CKD was determined by the calculation from the modification of diet in renal disease (MDRD) [21]: eGFR (mL/min per 1.73 m^2^) = 186 × SCr^−1.154^ × Age^−0.203^ × 0.742 (0.742 instead of 1.0, in the case of female). All comorbid conditions were screened as possible, including cardiovascular and cerebrovascular disease (cerebrovascular attack, coronary artery disease, peripheral arterial disease, and acute myocardial infarction), metabolic disease (hypertension and diabetes mellitus), liver disease (cirrhosis, peritonitis, ascites, and hepatoma), gastrointestinal bleeding, malignancy (lung and colon cancer), and shock. Prescriptions were all screened for angiotensin II receptor blockers or angiotensin-converting enzyme inhibitors, non-steroidal anti-inflammatory drugs, aspirin, aminoglycoside, statins, loop diuretics, steroids, and H_2_-blockers (famotidine and ranitidine).

### 2.3. Outcome Retrieval and Definitions

We set primary outcome as CA-AKI, which was defined according to the KDIGO practice guideline [12]: after CCT, an increase in SCr ≥ 0.3 mg/dL above baseline levels within 48 h or ≥ 50% within 7 days. To broaden the period of renal dysfunction to 7 days from 48–72 h, we chose not to follow the classical definition of CIN (i.e., an increase of SCr > 44 μmol/L or 25%, within 48–72 h following IV CM [11]). Because urine volumes were not collected periodically, we thus cannot include urine volume as a criterion for CI-AKI, as that was in the guideline of KDIGO.

The secondary endpoint was the necessity of evolving hemodialysis (i.e., dialysis-required AKI) within 30 days after CCT (the first documented hemodialysis within 30 days later than CCT). All indications and timing for urgent hemodialysis included refractory fluid excess even with diuretic drugs, severe hyperkalemia (>6.5 meq/L) even following potassium-lowering agents, >100 mg/dL of blood urea nitrogen, >6 mg/dL of SCr, metabolic acidosis (pH < 7.2), uremic encephalopathy, uremic bleeding, and uremic pericarditis. Mortality was also recorded for post-contrast complication.

We divided all patients to five groups according to stages of CKD [21]. In addition, the Youden index was used to establish the cutoff value of baseline SCr and eGFR to predict CA-AKI and dialysis within 30 days after the administration of CM.

This study was approved by Ethics Committee of Taichung Veterans General Hospital, IRB number: F15059. All patients’ data were retrospectively analyzed. According to the regulation, no patient’s informed consents were needed.

### 2.4. Statistical Analyses

Quantitative data are presented as mean ± standard deviation. Nominal and categorical variables were compared pairwise using the chi-square likelihood ratio or Fisher exact test with Bonferroni post hoc analyses. Continuous variables were evaluated by applying the nonparametric Wilcoxon test. We used univariate and multivariate logistic regression analyses to determine the associations between patient-related characteristics or comorbidities with CA-AKI and dialysis within 30 days and mortality. We used odds ratio (OR) and 95% confidence interval (CI) to determine the associations with the risks of CA-AKI and dialysis within 30 days after CCT. Two-sided *p*-values < 0.05 were considered statistically significant differences. We used the SPSS software (Statistical Package for the Social Science, version 20.0, Armonk, NY, USA) for all our statistical analyses.

## 3. Results

**Figure 1:**
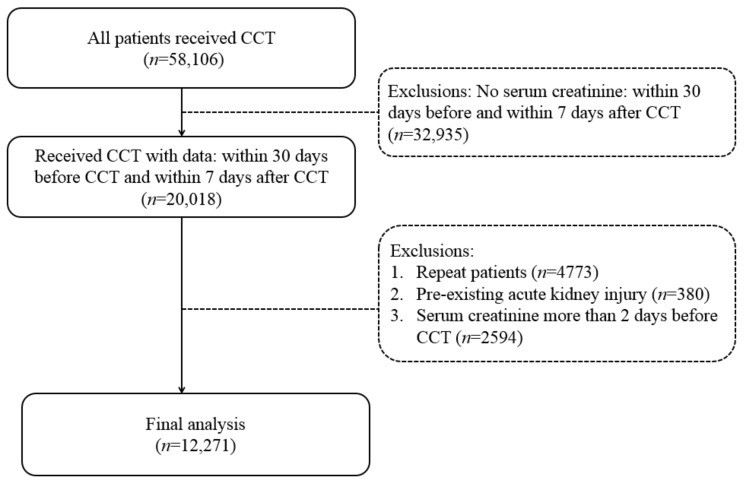
Patients selection flow chart.

The initial population undergoing CCT was 58,106 patients. After exclusion (no SCr data, repeated patients, and pre-existing AKI), 12,271 were finally included in this study (Figure 1). Their baseline data are shown in Table 1 (listed separately according to the five stages of CKD). Patients of later stages of CKD had more of the following: DM (*p* < 0.001), CAD (*p* < 0.001), AMI (*p* < 0.001), and gastrointestinal hemorrhage (*p* < 0.001). They also had higher serum levels of potassium (*p* < 0.001), fewer medications with NSAIDs (*p* < 0.001), more medications with aspirin (*p* < 0.001), and fewer using aminoglycosides (*p* < 0.001).

**Figure 2:**
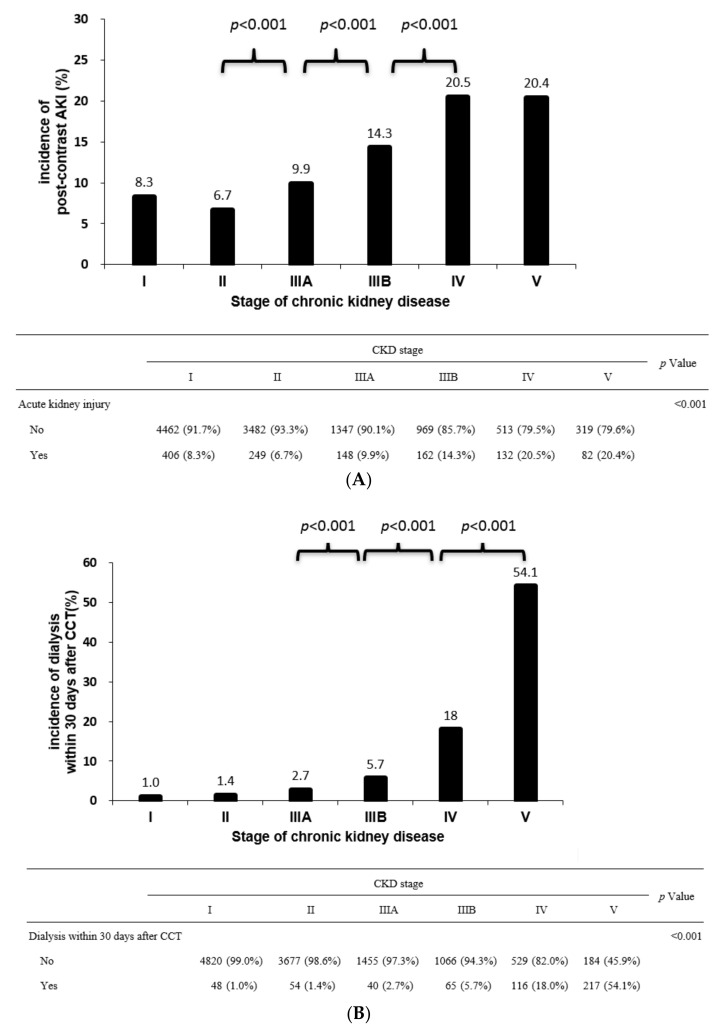

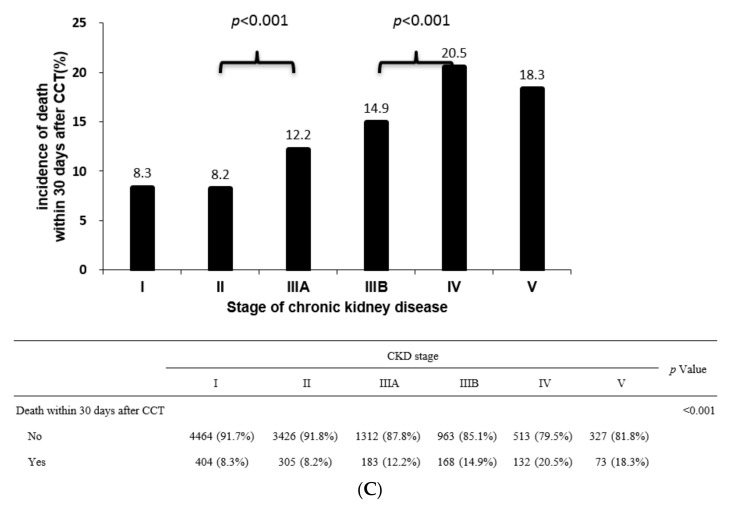
Outcomes of renal function outcomes according to baseline stages of CKD. All the three renal outcomes are significantly worse beyond the baseline renal function of stage IIIA. (**A**) Incidences of acute kidney injury after CCT according to baseline stages of CKD. Incidences are significant (*p* < 0.001) between stages I and IIIB–V; between stages II and IIIA to V; between stages IIIA and IIIB to V; and between stages IIIB and V. The increases in incidence are statistically significant starting from stage IIIA onward. Acute kidney injury after contrast computed tomography shown according to baseline stages of CKD; post hoc analysis showed *p* < 0.001 in I vs. IIIB, I vs. IV, I vs. V, II vs. IIIA, II vs. IIIB, II vs. IV, II vs. V, IIIA vs. IIIB, IIIA vs. IV, IIIA vs. V, and IIIB vs. IV. (**B**) Incidences of dialysis within 30 days after CCT shown according to baseline stages of CKD. The incidence was significantly different (*p* < 0.001) between stages I and IIIA to V; between stages II and IIIB to V; between stages IIIA and IIIB to V; between stages IIIB and IV to V; between stages IV and V. The incidence significantly increased from stage IIIA onward. Dialysis within 30 days after contrast computed tomography shown according to baseline stages of CKD, Post hoc analysis showed *p* < 0.001 in I vs. IIIA, I vs. IIIB, I vs. IV, I vs. V, II vs. IIIB, II vs. IV, II vs. V, IIIA vs. IIIB, IIIA vs. IV, IIIA vs. V, IIIB vs. IV, IIIB vs. V, and IV vs. V. (**C**) Incidences of death within 30 days after CCT shown according to baseline stages of CKD. The incidence was significantly different (*p* < 0.001) between stages I and IIIA to V; between stages II and s IIIA to V; between stages IIIA and IV to V; and between stages IIIB and IV. The incidence significantly increased from stage IIIA onward. Deaths within 30 days after contrast computed tomography shown according to baseline stages of CKD. Post hoc analysis showed *p* < 0.001 in I vs. IIIA, I vs. IIIB, I vs. IV, I vs. V, II vs. IIIA, II vs. IIIB, II vs. IV, II vs. V, IIIA vs. IV, IIIA vs. V, and IIIB vs. IV; chi-square test, ** *p* < 0.01.

**Figure 3:**
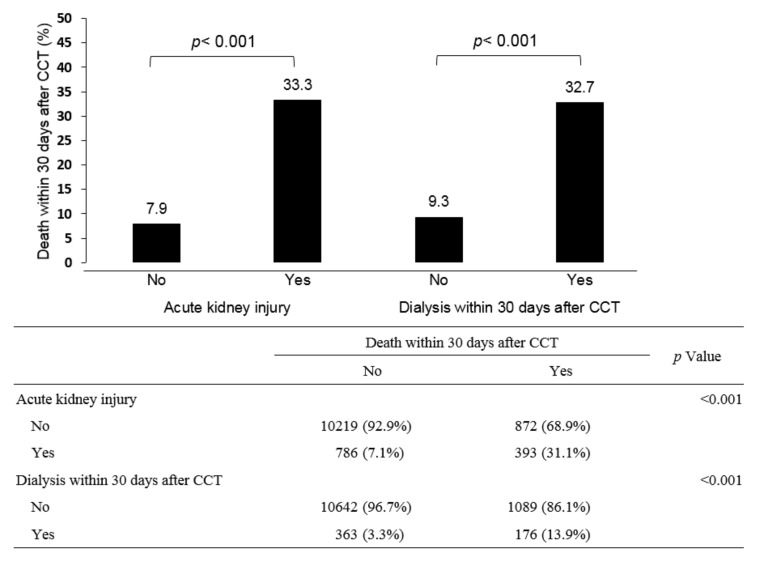
Once patients had developed post-CCT AKI, they more likely died (33.3% vs. 7.9%, *p* < 0.001). Similarly, once patients started with dialysis after CCT, they more likely died (32.7% vs. 9.3%, *p* < 0.001). The association between mortality within 30 days and AKI or dialysis within 30 days. Chi-square test, ** *p* < 0.01.

The associations between outcome after CM exposure and different stages of CKD are plotted in Figure 2A (for CA-AKI), Figure 2B (for dialysis-required AKI, within 30 days), and Figure 2C (for mortality within 30 days). CA-AKI incidence increased significantly starting from CKD stage 3A onward (*p* < 0.001) (Figure 2A). Incidences of CA-AKI were similar between stage 1 and stage 2 and also similar between stage 4 and stage 5 of CKD. In summary, the incidences of CA-AKI across CKD stages were as follows: stage 1 (8.3%) = stage 2 (6.7%) < stage 3A (9.9%) < stage 3B (14.3%) < stage 4 (20.5%) = stage 5 (20.4%). As for dialysis within 30 days (Figure 2B), such incidences increased significantly from CKD stage 3B onward. Incidences of dialysis within 30 days were as follows: stage 1 (1%) = stage 2 (1.4%) = stage 3A (2.7%) < stage 3B (5.7%) < stage 4 (18%) < stage 5 CKD (54.1%). Death within 30 days after CM increased significantly from CKD stage 3A onward (Figure 2C). Incidences of mortality were as follow: stage 1 (8.3%) = stage 2 (8.2%) < stage 3A (12.2%) = stage 3B (14.9%) < stage 4 (20.5%) = stage 5 (18.3%). Once with the occurrence of CA-AKI (7.9% vs. 33.3%, *p* < 0.001) or dialysis within 30 days (9.3% vs. 32.7%, *p* < 0.001), mortality rates increased significantly (Figure 3).

**Figure 4:**
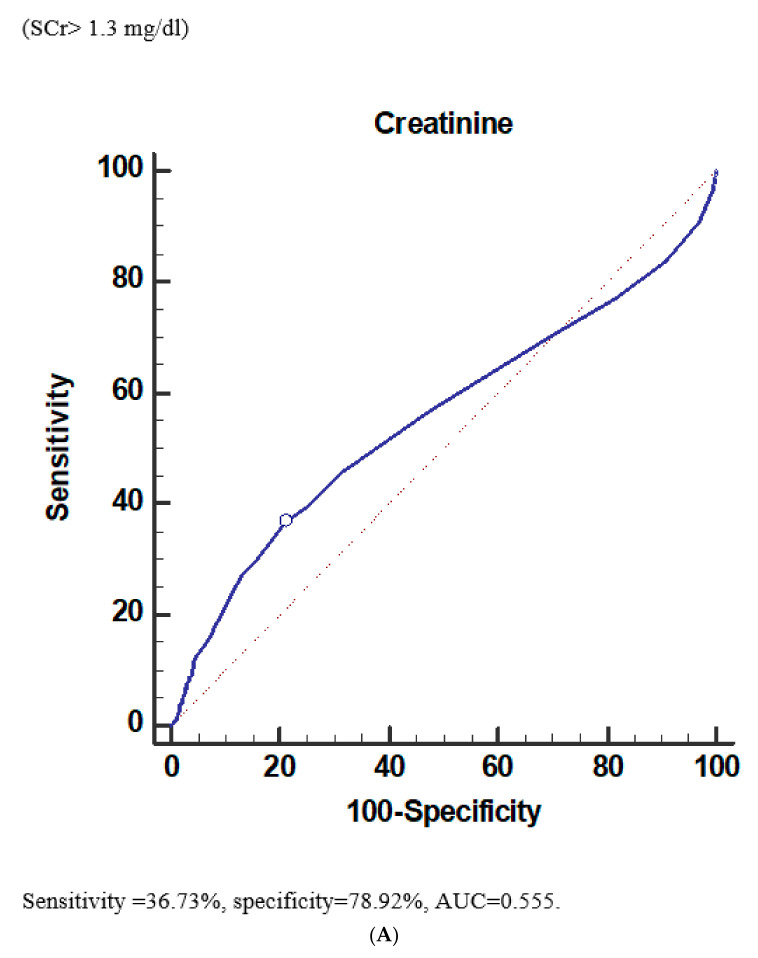

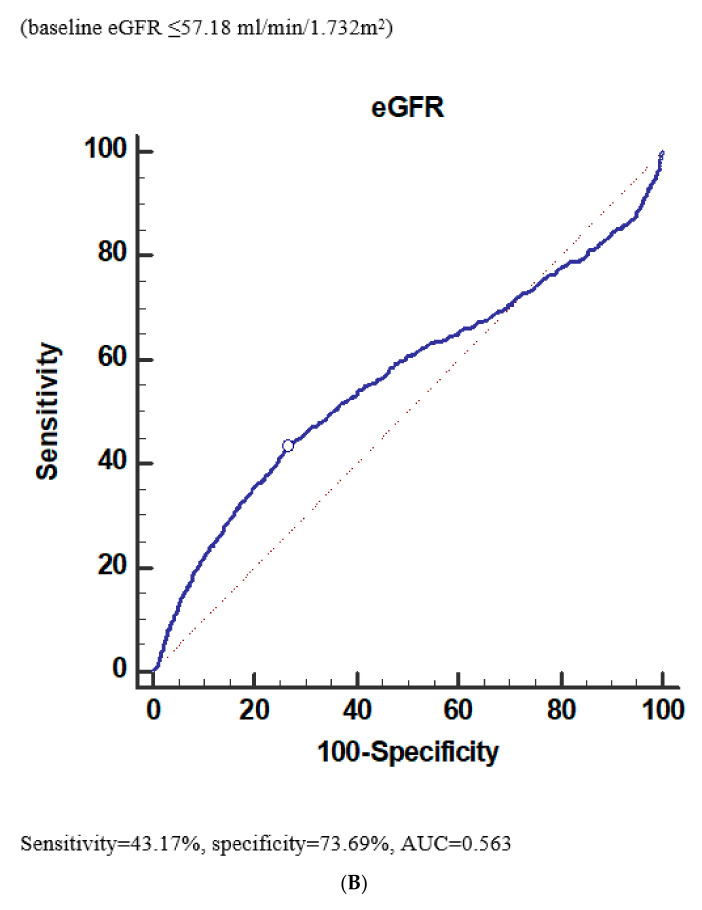

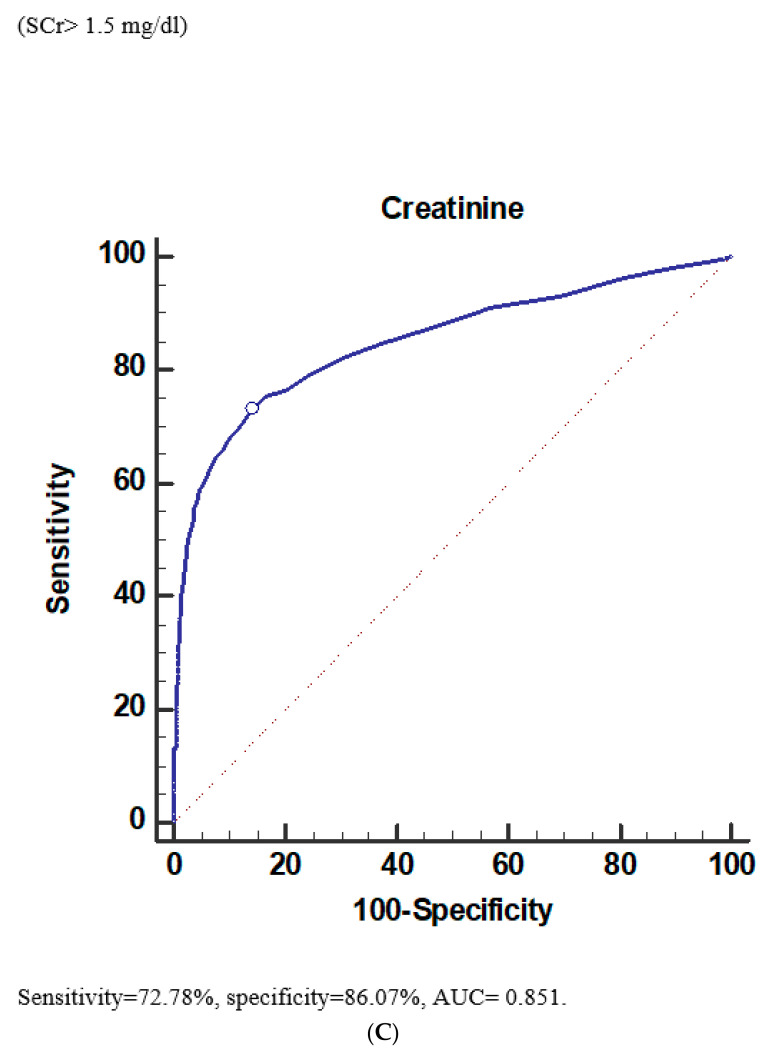

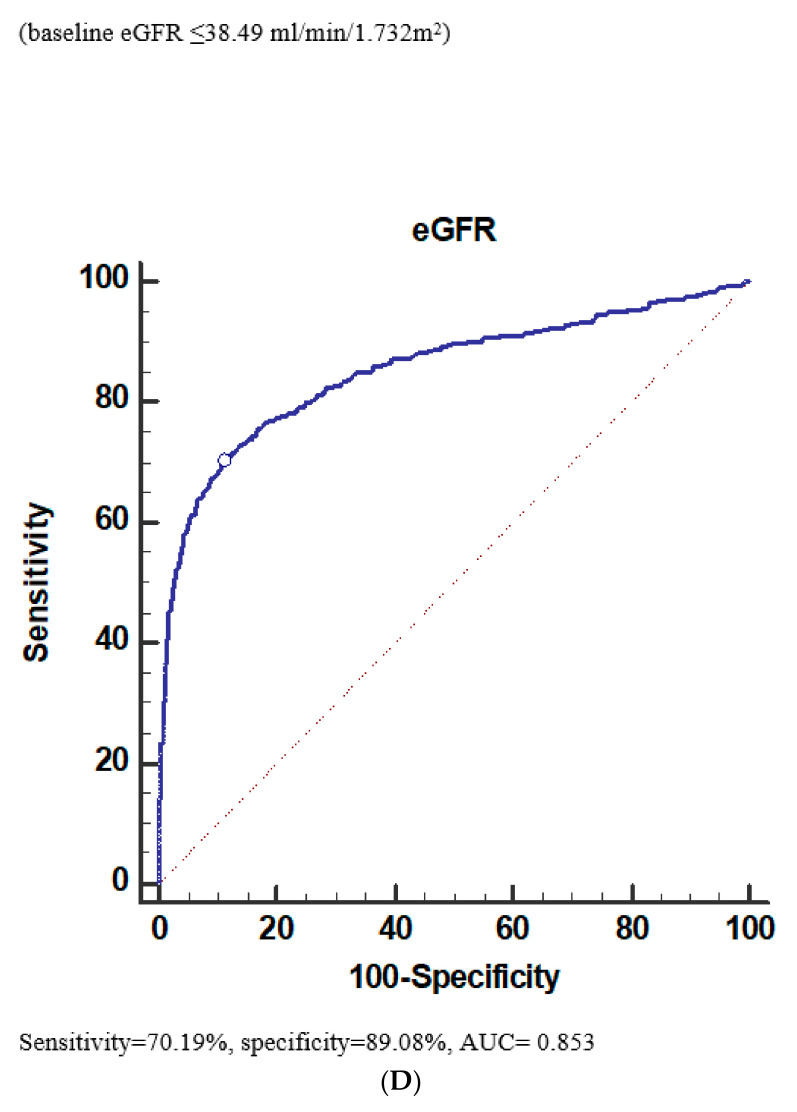
The predictive value of baseline serum creatinine (SCr) and eGFR to acute kidney injury and dialysis within 30 days after CCT. (**A**) The baseline serum creatinine (SCr) (>1.3 mg/dL) to predicting post-CCT AKI showed rather low sensitivity (36.73%) and high specificity (78.92%). The AUC was only 0.555. (**B**) The baseline eGFR (≤57.18 mL/min/1.732 m^2^) in predicting post-CCT AKI showed rather low sensitivity (43.17%) and high specificity (73.69%). The AUC was only 0.563. The baseline eGFR in predicting post-CCT AKI. (**C**) The baseline serum creatinine (SCr) (>1.5 mg/dL) in predicting dialysis within 30 days after CCT showed high sensitivity (72.78%) and high specificity (86.07%). The AUC was up to 0.851. The baseline serum creatinine (Scr) in predicting dialysis within 30 days after CCT. (**D**) The baseline eGFR (≤38.49 mL/min/1.732 m^2^) in predicting dialysis within 30 days after CCT showed high sensitivity (70.19%) and high specificity (89.08%). The AUC was up to 0.853. The baseline eGFR in predicting dialysis within 30 days after CCT.

The predictive power of baseline SCr > 1.3 mg/dL on CA-AKI was not good (36.73% of sensitivity, 78.92% of specificity, 0.555 of AUC) (Figure 4A). Similarly, predictive power of baseline eGFR ≤ 57.18 mL/min/1.732 m^2^ on CA-AKI was not good (43.17% of sensitivity, 73.69% of specificity, and 0.563 of AUC) (Figure 4B). On the other hand, the predictive powers were good on dialysis for baseline SCr > 1.5 mg/dL (72.78% sensitivity, 86.07% specificity, 0.851 of AUC) (Figure 4C) on dialysis and for baseline eGFR ≤ 38.49 mL/min/1.732 m^2^ (70.19% sensitivity, 89.08% specificity, and 0.853 of AUC) (Figure 4D).

In the multivariate Cox regression model, two independent risk factors were found for CA-AKI: stage 4 CKD (*p* = 0.001) and shock (*p* = 0.001) (Table 2). Similarly for dialysis, six independent risk factors were found: stage 3A (*p* = 0.010), stage 3B (*p* < 0.001), stage 4 (*p* < 0.001), and stage 5 CKD (*p* < 0.001), DM (*p* = 0.039), shock (*p* < 0.001), and longer prothrombin time (*p* = 0.019) (Table 3). As for mortality, nine independent risk factors were found: stage 3A (*p* = 0.01), stage 3B (*p* = 0.021), stage 4 (*p* < 0.001), and stage 5 (*p* = 0.004) CKD, hepatoma (*p* = 0.042), lung cancer (*p* < 0.001), shock (*p* < 0.001), and peritonitis (*p* = 0.026) (*p* < 0.001) (Table 4).

## 4. Discussion

Our present study is the biggest one of its kind in elucidating, after IV CM from CCT, the association between CA-AKI (rather than CIN) and different stages of CKD (with some difference between stage 4 and 5 CKD). Little previous study has provided clinicians the information regarding different incidences if any of CA-AKI in stage 4 and 5 CKD. Only one study mentioned the association of CA-AKI, dialysis, and mortality after CM administration between stage 4 and stage 5 CKD but with small numbers of patients (only 78 patients with stage 4–5 CKD) [10]. Two previous studies [4,5,6] showed that in CKD patients, the occurrence of CIN is 8% in stage 3A, 13% in stage 3B, and 27% in stages 4 and 5. The other three studies on CKD did not differentiate risks of CIN between stage 4 and 5 patients [18,22,23]. In CMSC of the ESUR, CIN is discussed on CKD but without differentiating stage 4 and 5 patients [13,17]. The major finding of our present study is that information is now available to medical professionals regarding incidence of CA-AKI in both stage 4 and stage 5 CKD patients. Compared to previous studies (on CIN rather than CA-AKI), the incidence of CA-AKI is 9.9% in stage 3A, 14.3% in stage 3B, 20.5% in stage 4, and 20.4% in stage 5 CKD (similar to stage 4 CKD). Comparing between CIN [4,5,6] and CA-AKI in CKD patients, their incidences are similar in stage 3A (8% vs. 9.9%) and stage 3B (13% vs. 14.3%). The incidence of CIN in stage 4–5 is 27%, and the incidence of CA-AKI is 20.4% in stage 4 and 20.5% in stage 5 CKD patients. In summary, the incidence of CA-AKI was similar to the incidence of CIN. CIN is defined as renal dysfunction within 48–72 h after CM exposure, but the AKI definition broadens the timeline up to 7 days after CM administration. However, with contrast-related renal dysfunctions, SCr levels almost all increased within 24 to 48 h after the CM exposure [24,25]. In our opinion, extending observation period to 7 days therefore offered little advantage for the evaluation of renal dysfunction.

Incidences of CA-AKI in stage 4 and 5 were nearly identical (20.4 vs. 20.5%), which did not indicate the same risk of renal function deterioration between stage 4 and 5 CKD after CM administration. First, increased SCr is not linearly related to the renal injury. The diagnosis of AKI based on KDIGO definition [12] likely underestimates the importance of real renal function injury in the severe stages of CKD. Similarly, in another study (based on new AKI definition), the incidence of CA-AKI was 10.3% in stage 4 CKD and 25% in stage 5 CKD [10]. The incidence did not increase significant from stage 4 to 5 CKD. Those will underestimate the importance of renal dysfunction between stage 4 and 5 CKD. In that study [10], they also excluded patients with SCr > 4 mg/dL, which is very severe selection bias. They excluded the highest risk for the requirement of dialysis. Only 78 patients of CKD stage 4–5 received contrast, but up to 576 patients of CKD stage 1–3 did. Therefore, the risk of renal dysfunction is not as low as that study in CKD stage 5 [10].

CA-AKI is a SCr-based analysis, but the dialysis-required AKI is a multiple clinical-variables-based outcome, which is a better marker for renal injury in the advanced stages of CKD. In clinical practice, the necessity for dialysis (clinical outcome) is more important than CA-AKI (laboratory outcome) to both patients and clinicians. The incidence of urgent hemodialysis markedly higher in stage 5 CKD compared to stage 4 CKD (51.4% vs. 18.0%, *p* < 0.001) even at similar incidences of CA-AKI. For dialysis-requiring AKI, a patient started at a lower GFR prior to AKI (more severe stage of CKD) would be more likely to begin dialysis than a patient started at a higher GFR (less severe stage of CKD). In this cohort, the predictive value of baseline SCr and eGFR were both better for urgent dialysis (sensitivity = 72.78%, specificity = 86.07%, AUC= 0.851, by SCr; sensitivity = 70.19%, specificity = 89.08%, AUC= 0.853, by eGFR) than for CA-AKI. As a result, we suggest here that not only the incidence of CA-AKI (laboratory outcome) but also dialysis-requiring AKI (clinical outcome) should be included in the outcome analysis of renal dysfunction after CM administration.

In 2010, the exposure to iodinated CM was implicated as the third most common cause of AKI with concomitant increased risks of dialysis and death [19]. The most common cause of CIN is pre-existing CKD because of the less-preserved renal function. Moreover, it is known that CIN in CKD patients have higher mortality rates compared to those without CKD [26]. However, the risk of CIN caused by CM administration may be overestimated [13,14]. There are hosts of confounding factors when addressing this issue: baseline condition, IA or IV CM, contrast volume, baseline conditions for IA (unstable hemodynamics, easier thromboli or emboli), types of CM (hyper-osmolality, hypo-osmolality, and isotonic CM), and first- or second-pass renal exposure. The type and volume of CM do matter. In the current era, low-osmolality CM agents cause fewer cases of CIN [15]. In this study, we only focused on the pure non-ionic iso-osmolar CM, iodixanol (Visipaque, Chicago, IL, USA), at a fixed volume (100 mL) to avoid bias. Second, CIN is more likely to occur in IA CM following cardiac angiography. The increased SCr may be due to background fluctuations of GFR that are common in sick, hospitalized patients. Patients receiving IA cardiac angiography may need volumes > 100 mL CM because of intervention and their less-stable hemodynamics. Reports on CIN are typically those cases following cardiac angiography. According to the recommendations of CMSC of the ESUR, eGFR levels < 45 mL/min/1.73 m^2^ before IV CM and eGFR levels < 60 mL/min/1.73 m^2^ before IA CM administrations represent a risk factor for CIN [16]. This same guideline in 2018 [13,17] suggested that if eGFR levels are < 45 mL/min/1.73 m^2^ and IA CM with first-pass renal exposure in the intensive care unit, prevention was suggested (evidence C). The prevention of CIN is therefore more likely after IA CM than IV CM [27,28]. Lower volumes and greater dilution before reaching the kidneys more likely happen in the case of IV CM. For IA CM, a score system has been established for CIN based on IA CM [28]. Renal injury after IV CM administration remains without consensus. Two meta-analyses in 2018 [10,29] reported no association between IV CM administration and CIN. Other studies also suggested that CIN after CCT may have been overlooked [18,23,30,31,32]. A retrospective study over a period of 7 years on 11,588 patients who underwent CT (5790 with CM, 7484 without) also supported similar results [33]. They considered CIN cannot be reflexively attributed to CM. However, in a propensity-matched analysis in 2019 [34], the administration of both IV and IA CM were found to associate with a risk of CIN. In our study, we did not have IA CM group for comparison, so we were uncertain the difference of risk of CA-AKI between IA and IV CM administration. However, in this cohort, the incidence of CA-AKI and dialysis-required AKI both increased at the worse baseline renal function.

A study by Bruce [30] suggested that the incidence of SCr elevation in patients receiving non-CM CCT was statistically similar to that in the iso-osmolar CM regardless of their baseline SCr levels and stages of CKD. However, in his study, only 62 (7.6%) patients with stage 4 and 5 CKD received IV CM CCT, and up to 1131 (15.1%) patients with stage 4 and 5 CKD received non-CM CT. Another 2015 study on CKD patients [18] reported that IV CM administration is not associated with an increased risk of CIN, emergent dialysis, and short-term mortality. However, their study had only a small number of patients with severe CKD (6% of stage 3 CKD and 17% of stage 4 and 5 CKD). Selection bias likely took place due to the fact that the sample size with severe CKD was small. Furthermore, those studies did not have same type or fixed volume of CM. However, in our study, we had more (1046) patients with stage 4 and 5 CKD receiving CCT with fixed contrast volume and had different baseline comorbidities. In other better-designed studies on IV CM after CCT, lower baseline eGFR was found to associate with more CIN [19,20,22,23]. Davenport et al. [22] reported that IV CM is a nephrotoxic risk factor for patients with eGFR levels consistently < 30 mL/min/1.73 m^2^, with a trend toward significance at 30–44 mL/min/1.73 m^2^. That is consistent with our finding that stage 4 CKD is associated with CA-AKI (OR = 1.90, 95%CI = 1.29–2.79, *p* < 0.001). Further, the baseline renal function (SCr and eGFR) can predict the dialysis-required CA-AKI with sufficient accuracy. Higher mortality likely occurs once with CA-AKI or dialysis after IV CM administration.

There are limitations of this study. First, we had no control group in this study. Second, this is a retrospective and single-center study. Third, we did not record the underlying condition of sepsis [35] in this study. However, we already included many comorbidities in this study, including DM, hypertension, CVA, PAOD, cirrhosis, hepatoma, colon cancer, lung cancer, AF, CAD, AMI, shock, peritonitis, ascites, and GI hemorrhage. Fourth, not every possible medication [36,37] and Chinese herbal medicine [38] affecting renal function was recorded in this study. However, we already included as many medications as possible, such as NSAID, aspirin, COX2i, aminoglycoside, loop diuretics, ACEi, ARB, steroid, statin, ranitidine, and famotidine. Fifth, we did not collect other biomarkers to correlate the association of CI-AKI [39], such as KIM-1, NGAL, IL-18, MCP-1, UMOD, and YKL-40. We also did not correlate new technology for the CI-AKI in this study, such as nanomedicine [40]. Finally, the recruitment period was long with the possibility of practices change over time.

## 5. Conclusions

Even without consensus on the renal toxicity of IV CM, we would like to suggest that the procedure of IV CM administration should be avoided for patients with severe stages of CKD to minimize outcomes of CA-AKI (another definition rather than CIN), dialysis, and mortality. Baseline SCr and eGFR both had good predictive power for dialysis-required AKI. Stage 4 and 5 CKD have the same risk for CA-AKI, but stage 5 CKD has markedly higher risk for dialysis.

## Figures and Tables

**Table 1:** Baseline characteristics of all participants divided according to their stages of CKD.

	CKD Stage												Total (*n* = 12,271)		*p*-Value
	I		II		IIIA		IIIB		IV		V				
Gender (Female)	2870	(59.0%)	2476	(66.4%)	971	(64.9%)	704	(62.2%)	367	(56.9%)	209	(52.1%)	7597	(61.9%)	<0.001
Age, years	57.13	±16.38	66.80	±15.14	72.02	±13.01	73.90	±13.41	73.65	±13.07	69.43	±13.89	64.70	±16.49	<0.001
Types of patients															<0.001
Outpatients	114	(2.3%)	89	(2.4%)	31	(2.1%)	21	(1.9%)	6	(0.9%)	7	(1.7%)	268	(2.2%)	
Inpatients	1971	(40.5%)	1388	(37.2%)	455	(30.4%)	311	(27.5%)	154	(23.9%)	111	(27.7%)	4390	(35.8%)	
Emergent room	2783	(57.2%)	2254	(60.4%)	1009	(67.5%)	799	(70.6%)	485	(75.2%)	283	(70.6%)	7613	(62.0%)	
Comorbidity															
DM	966	(19.8%)	1021	(27.4%)	566	(37.9%)	507	(44.8%)	311	(48.2%)	206	(51.4%)	3577	(29.2%)	<0.001
Hypertension	1641	(33.7%)	1928	(51.7%)	953	(63.7%)	776	(68.6%)	419	(65.0%)	268	(66.8%)	5985	(48.8%)	<0.001
CVA	522	(10.7%)	621	(16.6%)	291	(19.5%)	252	(22.3%)	146	(22.6%)	84	(20.9%)	1916	(15.6%)	<0.001
PAOD	65	(1.3%)	104	(2.8%)	49	(3.3%)	70	(6.2%)	33	(5.1%)	31	(7.7%)	352	(2.9%)	<0.001
Cirrhosis	514	(10.6%)	383	(10.3%)	187	(12.5%)	156	(13.8%)	87	(13.5%)	41	(10.2%)	1368	(11.1%)	0.002
Hepatoma	435	(8.9%)	315	(8.4%)	149	(10.0%)	114	(10.1%)	65	(10.1%)	27	(6.7%)	1105	(9.0%)	0.147
Colon cancer	477	(9.8%)	367	(9.8%)	127	(8.5%)	111	(9.8%)	50	(7.8%)	28	(7.0%)	1160	(9.5%)	0.149
Lung cancer	882	(18.1%)	725	(19.4%)	278	(18.6%)	146	(12.9%)	74	(11.5%)	32	(8.0%)	2137	(17.4%)	<0.001
AF	229	(4.7%)	341	(9.1%)	204	(13.6%)	175	(15.5%)	128	(19.8%)	65	(16.2%)	1142	(9.3%)	<0.001
CAD	404	(8.3%)	635	(17.0%)	364	(24.3%)	331	(29.3%)	204	(31.6%)	130	(32.4%)	2068	(16.9%)	<0.001
AMI	82	(1.7%)	144	(3.9%)	95	(6.4%)	94	(8.3%)	85	(13.2%)	59	(14.7%)	559	(4.6%)	<0.001
Shock	89	(1.8%)	78	(2.1%)	29	(1.9%)	51	(4.5%)	44	(6.8%)	12	(3.0%)	303	(2.5%)	<0.001
Peritonitis	101	(2.1%)	78	(2.1%)	32	(2.1%)	34	(3.0%)	25	(3.9%)	19	(4.7%)	289	(2.4%)	<0.001
Ascites	75	(1.5%)	40	(1.1%)	24	(1.6%)	15	(1.3%)	7	(1.1%)	9	(2.2%)	170	(1.4%)	0.245
GI hemorrhage	229	(4.7%)	197	(5.3%)	101	(6.8%)	103	(9.1%)	74	(11.5%)	51	(12.7%)	755	(6.2%)	<0.001
Laboratory data of blood															
Hb (*n* = 12,242)	12.30	±2.45	12.36	±2.49	11.98	±2.58	11.56	±2.67	11.05	±2.75	10.13	±2.72	12.08	±2.57	<0.001
Albumin (*n* = 6940)	3.42	±0.74	3.44	±0.73	3.31	±0.72	3.18	±0.72	3.00	±0.73	3.07	±0.67	3.35	±0.74	<0.001
Calcium (*n* = 9455)	7.81	±1.71	8.00	±1.70	8.06	±1.62	7.93	±1.78	7.92	±1.67	7.80	±1.86	7.91	±1.71	<0.001
Uric acid (*n* = 730)	4.98	±2.09	6.42	±2.60	7.26	±2.11	8.34	±2.94	8.73	±3.07	8.38	±2.61	6.67	±2.81	<0.001
Sodium (*n* = 12,153)	137.36	±5.37	137.91	±5.37	137.42	±6.19	137.56	±6.79	138.07	±7.56	137.20	±6.72	137.59	±5.80	<0.001
Potassium (*n* = 12,146)	3.98	±0.64	4.03	±0.65	4.10	±0.75	4.23	±0.87	4.34	±0.90	4.46	±1.02	4.07	±0.72	<0.001
pH (*n* = 9348)	6.89	±0.80	6.82	±0.86	6.82	±0.89	6.92	±0.85	7.01	±0.81	7.27	±0.56	6.89	±0.83	<0.001
HCO_3_^−^ (*n* = 5768)	25.14	±4.44	24.55	±4.82	23.68	±5.13	22.55	±5.54	20.31	±5.77	20.51	±5.89	23.84	±5.22	<0.001
PT (*n* = 9863)	11.49	±3.21	11.79	±5.41	12.00	±4.70	12.91	±7.26	13.58	±7.50	12.80	±6.77	11.93	±5.04	<0.001
INR (*n* = 9857)	1.09	±0.24	1.12	±0.49	1.15	±0.46	1.23	±0.67	1.30	±0.74	1.20	±0.49	1.14	±0.45	<0.001
Medications															
NSAID	2788	(57.3%)	1858	(49.8%)	608	(40.7%)	427	(37.8%)	204	(31.6%)	91	(22.7%)	5976	(48.7%)	<0.001
ASPIRIN	561	(11.5%)	749	(20.1%)	391	(26.2%)	337	(29.8%)	201	(31.2%)	130	(32.4%)	2369	(19.3%)	<0.001
COX2i	541	(11.1%)	460	(12.3%)	231	(15.5%)	154	(13.6%)	74	(11.5%)	38	(9.5%)	1498	(12.2%)	<0.001
Aminoglycoside	2285	(46.9%)	1651	(44.3%)	590	(39.5%)	407	(36.0%)	218	(33.8%)	114	(28.4%)	5265	(42.9%)	<0.001
Loop diuretics	2230	(45.8%)	1921	(51.5%)	946	(63.3%)	816	(72.1%)	497	(77.1%)	251	(62.6%)	6661	(54.3%)	<0.001
ACEI	258	(5.3%)	347	(9.3%)	199	(13.3%)	175	(15.5%)	96	(14.9%)	56	(14.0%)	1131	(9.2%)	<0.001
ARB	679	(13.9%)	726	(19.5%)	378	(25.3%)	371	(32.8%)	208	(32.2%)	134	(33.4%)	2496	(20.3%)	<0.001
Steroid	1022	(21.0%)	724	(19.4%)	314	(21.0%)	223	(19.7%)	129	(20.0%)	64	(16.0%)	2476	(20.2%)	0.125
Statin	264	(5.4%)	349	(9.4%)	196	(13.1%)	167	(14.8%)	103	(16.0%)	61	(15.2%)	1140	(9.3%)	<0.001
Ranitidine	439	(9.0%)	305	(8.2%)	113	(7.6%)	95	(8.4%)	55	(8.5%)	22	(5.5%)	1029	(8.4%)	0.131
Famotidine	869	(17.9%)	563	(15.1%)	211	(14.1%)	200	(17.7%)	109	(16.9%)	59	(14.7%)	2011	(16.4%)	0.001
BiPAP	202	(4.1%)	129	(3.5%)	56	(3.7%)	56	(5.0%)	53	(8.2%)	40	(10.0%)	536	(4.4%)	<0.001
ICU	466	(9.6%)	345	(9.2%)	164	(11.0%)	151	(13.4%)	135	(20.9%)	88	(21.9%)	1349	(11.0%)	<0.001
Sodium bicarbonate (*n* = 759)	145.58	±123.47	186.94	±221.86	156.15	±232.77	169.35	±188.41	200.99	±232.59	173.58	±151.50	175.69	±202.12	0.308
IV fluid > 1000.c.	802	(16.5%)	669	(17.9%)	265	(17.7%)	267	(23.6%)	212	(32.9%)	103	(25.7%)	2318	(18.9%)	<0.001
With Acetylcyestine	220	(4.5%)	194	(5.2%)	88	(5.9%)	78	(6.9%)	50	(7.8%)	17	(4.2%)	647	(5.3%)	<0.001

**Table 2:** Univariate and multivariate Cox regression analyses for the risk of acute kidney injury.

	Univariate			Multivariable		
	Odds Ratio	95%CI	*p*-Value	Odds Ratio	95%CI	*p*-Value
Age	1.01	(1.01–1.02)	<0.001	1.00	(0.99–1.01)	0.960
Gender						
F	Reference					
M	1.00	(0.88–1.13)	0.954			
CKD Stage						
I	Reference			Reference		
II	0.79	(0.67–0.93)	0.004	0.91	(0.67–1.22)	0.528
IIIA	1.21	(0.99–1.47)	0.062	1.19	(0.83–1.69)	0.346
IIIB	1.84	(1.51–2.23)	<0.001	1.42	(1.00–2.02)	0.053
IV	2.83	(2.28–3.51)	<0.001	1.90	(1.29–2.79)	0.001
V	2.83	(2.17–3.68)	<0.001	1.45	(0.95–2.22)	0.086
Comorbidity						
DM	1.60	(1.41–1.81)	<0.001	1.05	(0.84–1.32)	0.650
HT	1.35	(1.19–1.52)	<0.001	1.15	(0.91–1.46)	0.238
CVA	1.26	(1.07–1.47)	0.004	0.98	(0.75–1.29)	0.901
PAOD	1.32	(0.95–1.83)	0.093			
LC	1.33	(1.11–1.58)	0.002	0.79	(0.58–1.08)	0.141
Liver ca	1.30	(1.07–1.58)	0.008	1.02	(0.72–1.45)	0.899
Colon ca	0.90	(0.73–1.11)	0.323			
Lung ca	0.85	(0.72–1.01)	0.060			
AF	1.55	(1.29–1.86)	<0.001	1.11	(0.81–1.51)	0.514
CAD	1.48	(1.28–1.71)	<0.001	0.96	(0.72–1.29)	0.792
AMI	2.09	(1.67–2.62)	<0.001	1.18	(0.79–1.77)	0.426
Shock	3.81	(2.94–4.94)	<0.001	2.15	(1.53–3.02)	<0.001
Peritonitis	1.39	(0.98–1.98)	0.063			
Ascites	1.88	(1.25–2.83)	0.003	1.55	(0.82–2.91)	0.175
GI hemorrhage	1.53	(1.23–1.90)	<0.001	1.07	(0.78–1.48)	0.671
Laboratory data						
Hb	0.91	(0.89–0.93)	<0.001	0.98	(0.95–1.02)	0.390
Alb	0.52	(0.47–0.57)	<0.001	0.88	(0.75–1.04)	0.145
Ca	0.93	(0.90–0.97)	<0.001	0.92	(0.86–0.99)	0.025
UA	1.10	(1.02–1.19)	0.010			
NA	0.99	(0.98–1.00)	0.045	1.00	(0.99–1.01)	0.969
K	1.16	(1.07–1.26)	<0.001	1.12	(0.99–1.27)	0.071
pH	1.32	(1.21–1.44)	<0.001	1.18	(0.98–1.42)	0.073
HCO_3_^−^	0.97	(0.95–0.98)	<0.001	0.99	(0.98–1.01)	0.567
PT	1.03	(1.02–1.04)	<0.001	1.02	(0.87–1.20)	0.821
INR	1.43	(1.29–1.59)	<0.001	0.87	(0.16–4.59)	0.866

Logistic regression. * *p* < 0.05, ** *p* < 0.01.

**Table 3:** Univariate and multivariate Cox regression analyses for the risk of dialysis within 30 days after CCT.

	Univariate			Multivariable		
	Odds Ratio	95%CI	*p*-Value	Odds Ratio	95%CI	*p*-Value
Age	1.02	(1.01–1.02)	<0.001	0.99	(0.98–1.00)	0.102
Gender						
F	Reference					
M	0.90	(0.75–1.07)	0.228			
CKD Stage						
I	Reference			Reference		
II	1.47	(1.00–2.18)	0.052	1.69	(1.00–2.86)	0.052
IIIA	2.76	(1.81–4.22)	<0.001	2.19	(1.20–4.00)	0.010
IIIB	6.12	(4.19–8.94)	<0.001	3.98	(2.32–6.81)	<0.001
IV	22.02	(15.55–31.19)	<0.001	11.06	(6.58–18.58)	<0.001
V	118.43	(83.83–167.31)	<0.001	36.38	(21.58–61.34)	<0.001
Comorbidity						
DM	2.41	(2.03–2.87)	<0.001	1.36	(1.02–1.81)	0.039
HT	1.60	(1.34–1.90)	<0.001	0.90	(0.66–1.22)	0.488
CVA	1.39	(1.12–1.72)	0.003	1.08	(0.75–1.53)	0.688
PAOD	2.67	(1.87–3.79)	<0.001	0.79	(0.38–1.64)	0.534
LC	1.52	(1.20–1.93)	0.001	0.99	(0.67–1.44)	0.941
Liver ca	1.06	(0.79–1.42)	0.715			
Colon ca	0.72	(0.51–1.00)	0.051			
Lung ca	0.41	(0.30–0.56)	<0.001	0.60	(0.36–1.00)	0.050
AF	2.03	(1.61–2.57)	<0.001	1.12	(0.75–1.65)	0.584
CAD	2.09	(1.72–2.53)	<0.001	1.05	(0.73–1.51)	0.809
AMI	2.99	(2.27–3.95)	<0.001	1.05	(0.64–1.71)	0.856
Shock	5.00	(3.67–6.81)	<0.001	2.16	(1.42–3.29)	<0.001
Peritonitis	3.13	(2.18–4.51)	<0.001	1.35	(0.79–2.31)	0.276
Ascites	1.97	(1.14–3.44)	0.016	1.68	(0.76–3.73)	0.200
GI hemorrhage	1.94	(1.47–2.58)	<0.001	0.82	(0.53–1.26)	0.359
Laboratory data						
Hb	0.79	(0.77–0.82)	<0.001	0.95	(0.90–1.00)	0.038
Alb	0.44	(0.38–0.50)	<0.001	0.66	(0.53–0.83)	<0.001
Ca	0.95	(0.91–1.00)	0.068			
UA	1.21	(1.11–1.33)	<0.001			
NA	0.99	(0.98–1.01)	0.332			
K	1.48	(1.34–1.64)	<0.001	1.12	(0.96–1.31)	0.140
PH	1.70	(1.48–1.96)	<0.001	0.99	(0.79–1.24)	0.918
HCO_3_^−^	0.90	(0.89–0.92)	<0.001	1.00	(0.98–1.03)	0.940
PT	1.03	(1.02–1.04)	<0.001	1.30	(1.04–1.61)	0.019
INR	1.38	(1.23–1.54)	<0.001	0.07	(0.01–0.69)	0.023

Logistic regression. * *p* < 0.05, ** *p* < 0.01.

**Table 4:** Univariate and multivariate Cox regression analyses for the risk of mortality within 30 days after CCT.

	Univariate			Multivariable		
	Odds Ratio	95%CI	*p*-Value	Odds Ratio	95%CI	*p*-Value
Age	1.02	(1.01–1.02)	<0.001	1.00	(1.00–1.01)	0.164
Gender						
F	Reference			Reference		
M	1.19	(1.05–1.34)	0.006	0.94	(0.77–1.15)	0.553
CKD Stage						
I	Reference			Reference		
II	0.98	(0.84–1.15)	0.835	1.28	(0.98–1.66)	0.067
IIIA	1.54	(1.28–1.85)	<0.001	1.50	(1.09–2.07)	0.013
IIIB	1.93	(1.59–2.34)	<0.001	1.47	(1.06–2.04)	0.021
IV	2.84	(2.29–3.53)	<0.001	2.00	(1.40–2.87)	<0.001
V	2.47	(1.88–3.24)	<0.001	1.79	(1.20–2.68)	0.004
Comorbidity						
DM	1.08	(0.96–1.23)	0.209			
HT	0.91	(0.81–1.03)	0.124			
CVA	0.84	(0.71–1.00)	0.045	0.77	(0.60–1.00)	0.054
PAOD	0.75	(0.50–1.10)	0.142			
LC	1.80	(1.53–2.10)	<0.001	0.90	(0.69–1.19)	0.471
Liver ca	2.07	(1.75–2.44)	<0.001	1.36	(1.01–1.83)	0.042
Colon ca	0.81	(0.66–1.01)	0.060			
Lung ca	2.02	(1.77–2.31)	<0.001	2.68	(2.10–3.42)	<0.001
AF	1.29	(1.07–1.55)	0.007	0.84	(0.62–1.14)	0.260
CAD	1.04	(0.89–1.21)	0.646			
AMI	1.48	(1.16–1.89)	0.002	1.22	(0.85–1.74)	0.288
Shock	6.77	(5.34–8.57)	<0.001	3.11	(2.28–4.23)	<0.001
Peritonitis	1.82	(1.33–2.48)	<0.001	0.61	(0.39–0.94)	0.026
Ascites	2.92	(2.05–4.16)	<0.001	1.47	(0.85–2.55)	0.172
GI hemorrhage	1.71	(1.40–2.10)	<0.001	0.94	(0.70–1.26)	0.685
Laboratory data						
Hb	0.83	(0.82–0.85)	<0.001	0.96	(0.93–1.00)	0.027
Alb	0.34	(0.30–0.37)	<0.001	0.54	(0.47–0.63)	<0.001
Ca	1.00	(0.97–1.04)	0.940			
UA	1.12	(1.04–1.20)	0.001			
NA	0.96	(0.95–0.97)	<0.001	0.99	(0.98–1.01)	0.367
K	1.14	(1.06–1.23)	0.001	1.08	(0.96–1.21)	0.193
PH	1.35	(1.24–1.47)	<0.001	1.01	(0.86–1.18)	0.932
HCO_3_	0.96	(0.95–0.97)	<0.001	0.99	(0.98–1.01)	0.416
PT	1.06	(1.05–1.07)	<0.001	1.00	(0.86–1.17)	0.965
INR	2.00	(1.76–2.27)	<0.001	1.26	(0.25–6.37)	0.782
BilT	1.12	(1.10–1.14)	<0.001	1.08	(1.05–1.10)	<0.001

Logistic regression. * *p* < 0.05, ** *p* < 0.01.

## Data Availability

Data cannot be shared due to the regulation of local institute.
